# A Contribution to Surgical Therapeutics

**Published:** 1882-08

**Authors:** John Hall

**Affiliations:** Toronto; Bureau of Surgical Therapeutics, I. H. A.


### A CONTRIBUTION TO SURGICAL THERAPEUTICS.

JOHN HALL, M. D., TORONTO.

(*Bureau of Surgical Therapeutics, I. H. A.*)

In the year 18—, a lady called on me for advice, giving the following history of her case: Three days after the birth of her second child, the lochia suddenly ceased, succeeded by severe lancinations in the right ovarian region, with some swelling of the part affected. The pain continued, more or less, from this time until the birth of her third child, from which she fairly recovered, though the ovarian pain never wholly ceased, and the part beginning to assume larger proportions, a necessity arose for further advice, and the late Dr. Bovell was consulted (at that time the most eminent physician in the city) who, after expressing fear that she had an ovarian tumor, suggested and brought about an examination by Drs. Beaumont, Lizars and R——, all men of high standing in their profession as surgeons, indeed of European reputation.

The result of their united consultation was a weekly visit to examine and watch the apparent growth; supplemented by a further consultation, in which the late Dr. Hodder and others were included, and from whom was at length obtained a unanimous decision that the patient was suffering from an ovarian tumor of some three years' growth—that the only resort was removal by the knife, and that at the present rate of increase, the said operation could not be safely deferred beyond six weeks. She had thus the prospect of an operation she dared not face, and was thereby driven to try Homœopathy, as those who proposed the operation would give no assurance of a favorable termination.

Under these circumstances she consulted me, as she said, "without the slightest hope of a cure, or faith in Homœopathy, but simply to feel that she was doing *something*."

My own examination yielded the following: Mrs. A——, aged thirty, mother of three children, moderate stature, well-developed muscles, dark-brown hair, *active and cheerful temperament. Tumor of right ovary*, of three years' duration, increasing in size and attended with almost *continuous grinding, wearying pain* and *sense of weight* which made her cry out, clench her teeth and hold her breath, while she *doubled herself up*, pressing the part, to try and numb the pain for a time, with only partial relief.

There would be seasons of complete rest during several hours, followed by a *sharper and more lancinating pain*, which by degrees gave place to the almost continuous *dull aching and grinding—menstrual flow profuse.*

My treatment consisted almost exclusively of *Apium virus*, which was continued at long intervals during a period of some nine months—the tumor reducing in size thereby about two-thirds, while the pain was but slightly relieved.

Finding much difficulty, at the end of this time, in eliciting anything further from my patient, whereby a new remedy could be diagnosed, I had desired her for a season to remit treatment, in hope that a few weeks might furnish some further development from which the treatment might be resumed with confidence, Apis having fairly exhausted its remedial action. This hope was not disappointed, for noticing one day with what *energy* and *rapidity she was walking*, I asked her how she could do so under so painful a malady, and was promptly answered, "*I feel a great* NECESSITY *to be* ALWAYS *on the move.* I cannot sit or stand or rest, *but seem as if I could walk forever,* and hardly *lie* still unless I am tired out with the incessant aching and fall asleep from sheer exhaustion." After a short remonstrance that so important a condition should have been withheld from me, as of no moment, my mind immediately recalled a symptom of *Fluoric acid,* as given in line No. 85 in Lippe's admirable Text-Book of Homœopathic Materia Medica, and since then reproduced in Hering's Condensed Materia Medica. The text runs thus: "*Increased ability to exercise his muscles without fatigue, regardless of the most excessive heat of summer or cold in winter.*" Here seemed a marked simillimum for a very peculiar symptom, but unfortunately there was not to be found in all the published provings of this drug any indication of ovarian action, excepting perhaps a hint in the too early and profuse menstruation. Could, then, a drug give hope of remedial power when, so far as our knowledge extended, it failed to show *any action on an organ corresponding to an acknowledged diseased condition of such organ;* in other words, the *name* of a disease given. Shall not such pathological name guide us in selecting our remedy for it? and if so, what possible benefit could result from Fluoric acid in *tumor* of the *right ovary* which, as stated before, contains no ovarian symptoms among the provings? We should find a satisfactory answer to these inquiries only in the pages of that much neglected, misunderstood, badly abused, but indispensable treasure of therapeutic knowledge, the *Organon* of Samuel Hahnemann, where the grand old man thus discourses in that inimitable paragraph 153, "In searching after a homœopathic specific remedy, in order to discover an artificial morbific power resembling the natural disease which is to be cured, we ought to be particularly and ALMOST EXCLUSIVELY ATTENTIVE to the symptoms that are *striking, singular, extraordinary* and *peculiar* (characteristic); for it is *to these latter that similar symptoms from among those created by the medicine ought to correspond* in order to constitute it the remedy most suitable to the case." Now, according to the profound teaching here given, the pathological name of a malady is nowhere, not even hinted at as a guide in the selection of our remedy, while the *striking, singular, ex-*

*traordinary, peculiar* and I will add INEXPLICABLE symptoms furnish the *full data* for accuracy and consequent success; in compliance, then, with this gratefully received instruction I gave my patient *Fluoric acid* in rarely repeated doses, and at the expiration of three months, one year from beginning the treatment, I had the satisfaction of finding all pain subsided and the tumor entirely removed, while all the various functions, excepting that of a profuse menstrual flow, have gone on healthily ever since (now 15 years).

The potencies then in my possession were 30ths, but I do not doubt that the very highest which have since been obtained and in daily use would have greatly expedited the cure.

Of true Hahnemannian Homœopathy then, it is safe to reiterate, that "by her medical treatment, cases are cured without the knife, where that mode of procedure in other schools is the only alternative, and numerous lives are being daily saved after capital operations and their consequent fevers, which in other places are as daily sacrificed where true Homœopathy is unknown."

Who can refrain from moralizing on the responsibility of being intrusted with so benign an art and what an awful reckoning is in store for those who suppress its radiant light for the misty uncertainties of modern therapeutics.

